# 2,3,5,6-Tetra­fluoro-1,4-bis­({[(thio­phen-2-yl)methyl­idene]amino}­meth­yl)benzene

**DOI:** 10.1107/S1600536814012434

**Published:** 2014-06-04

**Authors:** Ning Gao, Hai-Kun Luo, Rui-Rui Qin, Ming-Yang He

**Affiliations:** a School of Petrochemical Engineering, Changzhou University, Changzhou 213164, People’s Republic of China

## Abstract

In the title compound, C_18_H_12_F_4_N_2_S_2_, a bis-thio­phenyl Schiff base ligand with a perifluorinated aromatic core, the complete molecule is generated by crystallographic inversion symmetry. The thio­phene and tetra­fluorinated benzene rings are oriented at a dihedral angle of 77.38 (4)°. The crystal structure exhibits C—H⋯F hydrogen bonds, resulting in supra­molecular chains along the *c-*axis direction.

## Related literature   

For background information on thio­phene-based Schiff base ligands, see: Hee & Soon (2007 ▶); Fang *et al.* (2001 ▶). For fluorine-functionalized complexes, see Chen *et al.* (2012 ▶). Zhang *et al.* (2011 ▶) describe the synthesis of the title compound.
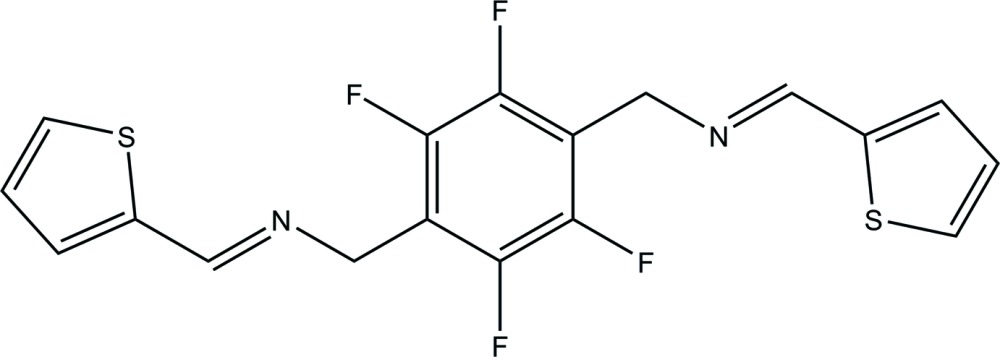


## Experimental   

### 

#### Crystal data  

C_18_H_12_F_4_N_2_S_2_
*M* *_r_* = 396.42
Monoclinic, 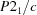

*a* = 15.500 (7) Å
*b* = 4.865 (2) Å
*c* = 11.713 (6) Å
β = 95.371 (9)°
*V* = 879.3 (7) Å^3^
*Z* = 2
Mo *K*α radiation
μ = 0.35 mm^−1^
*T* = 296 K
0.24 × 0.22 × 0.20 mm

#### Data collection  

Bruker SMART APEX CCD diffractometer
Absorption correction: multi-scan (*SADABS*; Sheldrick, 2003 ▶) *T*_min_ = 0.921, *T*_max_ = 0.934
6136 measured reflections
1541 independent reflections
1427 reflections with *I* > 2σ(*I*)
*R*_int_ = 0.018

#### Refinement  

*R*[*F* ^2^ > 2σ(*F* ^2^)] = 0.030
*wR*(*F* ^2^) = 0.090
*S* = 1.02
1541 reflections
118 parameters
H-atom parameters constrained
Δρ_max_ = 0.18 e Å^−3^
Δρ_min_ = −0.28 e Å^−3^

### 

Data collection: *APEX2* (Bruker, 2007 ▶); cell refinement: *APEX2* and *SAINT* (Bruker, 2007 ▶); data reduction: *SAINT* (Bruker, 2007 ▶); program(s) used to solve structure: *SHELXS97* (Sheldrick, 2008 ▶); program(s) used to refine structure: *SHELXL97* (Sheldrick, 2008 ▶); molecular graphics: *SHELXTL* (Sheldrick, 2008 ▶) and *DIAMOND* (Brandenburg, 2005 ▶); software used to prepare material for publication: *SHELXTL*.

## Supplementary Material

Crystal structure: contains datablock(s) I, 1. DOI: 10.1107/S1600536814012434/im2454sup1.cif

Structure factors: contains datablock(s) I. DOI: 10.1107/S1600536814012434/im2454Isup2.hkl

Supporting information file. DOI: 10.1107/S1600536814012434/im2454Isup3.cml

CCDC reference: 1005697

Additional supporting information:  crystallographic information; 3D view; checkCIF report

## Figures and Tables

**Table 1:** Hydrogen-bond geometry (Å, °)

*D*—H⋯*A*	*D*—H	H⋯*A*	*D*⋯*A*	*D*—H⋯*A*
C5—H5⋯F1^i^	0.93	2.56	3.446 (2)	159
C6—H6*B*⋯F1^i^	0.97	2.63	3.542 (2)	156

Symmetry code: (i) 
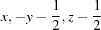
.

**Fig. 1.**
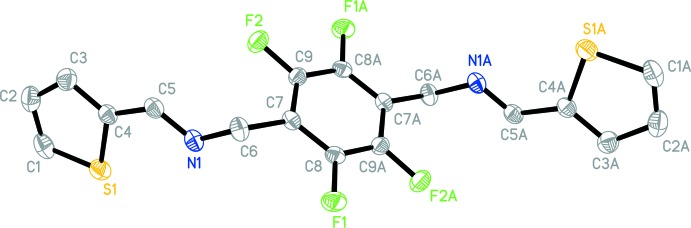
Molecular structure of the title compound with the atom-numbering scheme. Displacement ellipsoids are drawn at the 30% probability level. Hydrogen atoms are omitted for clarity.

**Fig. 2.**
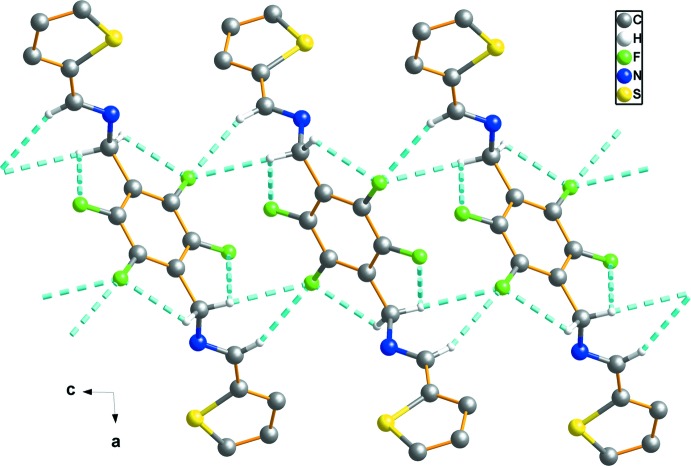
View of the one-dimensional supramolecular chain structure constructed via intermolecular C—H···F hydrogen-bonding interactions (irrelative hydrogen atoms are omitted for clarity).

## supplementary crystallographic information

### S1. Comment

In the past decade, thiophen-based bidentate Schiff base ligands have been utilized intensively to assemble various coordination compounds with intriguing structural features and potential applications (Hee & Soon, 2007; Fang *et al.*, 2001). As part of our ongoing studies of the effect of fluorine substitution on crystal structures of coordination polymers (Chen *et al.*, 2012), herein, we wish to report the crystal structure of the title compound, 2,3,5,6-tetrafluoro-1,4-bis(1-(1-thiophen-2-yl)methyleneaminomethyl)benzene, (I).

A perspective view of (I), including the atomic numbering scheme, is shown in Fig. 1. (I) crystallizes around a crystallographic centre with a half molecule in the asymmetric unit. Bond lengths and angles are within normal ranges. The terminal thiophenyl groups are coplanar, and they form a dihedral angle of 77.38 (4)° with the central tetrafluorinated benzene ring. Intermolecular C—H···F hydrogen-bonding interactions link the adjacent molecules to generate one-dimensional supramolecular chains along the *c* axis (see Fig. 2).

### S2. Experimental

Compound (I) was synthesized and purified according to the method described by Zhang *et al.* (2011) through a condensation of 2,3,5,6-tetrafluoro-1,4-benzenedimethanamine with thiophene-2-carboxaldehyde (yied 75%). ^1^H NMR (CDCl_3_): δ 2.71 (s, 4H, CH_2_), 7.19 (s, 2H, CH), 7.58 (s, 2H, CH), 7.84 (s, 2H, CH), 8.02 (s, 2H, CH).

Colourless needle-like single crystals (m.p. 452.3—452.6 K) suitable for X-ray analysis were obtained by dissolving (I) (20.0 mg) in dichloromethane (6 ml) solution and then slowly evaporating the solvent at room temperature for a period of about one week.

### S3. Refinement

All H atoms bound to carbon were assigned to calculated positions, with C—H = 0.97 Å (methylene) and 0.93 Å (aromatic and imine), and refined using a riding model, with *U*_iso_(H)=1.2U_eq_ (C).

### Crystal data

**Table:**

C_18_H_12_F_4_N_2_S_2_	*F*(000) = 404
*M**_r_* = 396.42	*D*_x_ = 1.497 Mg m^−^^3^
Monoclinic, *P*2_1_/*c*	Mo *K*α radiation, λ = 0.71073 Å
Hall symbol: -P 2ybc	Cell parameters from 5195 reflections
*a* = 15.500 (7) Å	θ = 2.6–31.7°
*b* = 4.865 (2) Å	µ = 0.35 mm^−^^1^
*c* = 11.713 (6) Å	*T* = 296 K
β = 95.371 (9)°	Shape, colourless
*V* = 879.3 (7) Å^3^	0.24 × 0.22 × 0.20 mm
*Z* = 2	

### Data collection

**Table:**

Bruker SMART APEX CCD diffractometer	1541 independent reflections
Radiation source: fine-focus sealed tube	1427 reflections with *I* > 2σ(*I*)
Graphite monochromator	*R*_int_ = 0.018
phi and ω scans	θ_max_ = 25.0°, θ_min_ = 2.6°
Absorption correction: multi-scan (*SADABS*; Sheldrick, 2003)	*h* = −18→17
*T*_min_ = 0.921, *T*_max_ = 0.934	*k* = −5→5
6136 measured reflections	*l* = −13→12

### Refinement

**Table:**

Refinement on *F*^2^	Primary atom site location: structure-invariant direct methods
Least-squares matrix: full	Secondary atom site location: difference Fourier map
*R*[*F*^2^ > 2σ(*F*^2^)] = 0.030	Hydrogen site location: inferred from neighbouring sites
*wR*(*F*^2^) = 0.090	H-atom parameters constrained
*S* = 1.02	*w* = 1/[σ^2^(*F*_o_^2^) + (0.0516*P*)^2^ + 0.297*P*] where *P* = (*F*_o_^2^ + 2*F*_c_^2^)/3
1541 reflections	(Δ/σ)_max_ = 0.001
118 parameters	Δρ_max_ = 0.18 e Å^−^^3^
0 restraints	Δρ_min_ = −0.28 e Å^−^^3^

### Special details

**Table:**

Geometry. All esds (except the esd in the dihedral angle between two l.s. planes) are estimated using the full covariance matrix. The cell esds are taken into account individually in the estimation of esds in distances, angles and torsion angles; correlations between esds in cell parameters are only used when they are defined by crystal symmetry. An approximate (isotropic) treatment of cell esds is used for estimating esds involving l.s. planes.
Refinement. Refinement of F^2^ against ALL reflections. The weighted R-factor wR and goodness of fit S are based on F^2^, conventional R-factors R are based on F, with F set to zero for negative F^2^. The threshold expression of F^2^ > 2sigma(F^2^) is used only for calculating R-factors(gt) etc. and is not relevant to the choice of reflections for refinement. R-factors based on F^2^ are statistically about twice as large as those based on F, and R- factors based on ALL data will be even larger.

### Fractional atomic coordinates and isotropic or equivalent isotropic displacement parameters (Å^2^)

**Table:**

	*x*	*y*	*z*	*U*_iso_*/*U*_eq_	
C1	0.43101 (11)	0.7020 (4)	0.85018 (19)	0.0605 (5)	
H1	0.4750	0.8166	0.8818	0.073*	
C2	0.40375 (12)	0.6938 (4)	0.73736 (18)	0.0610 (5)	
H2	0.4266	0.8036	0.6826	0.073*	
C3	0.33673 (12)	0.5000 (4)	0.71153 (16)	0.0531 (4)	
H3	0.3110	0.4671	0.6378	0.064*	
C4	0.31367 (10)	0.3652 (3)	0.80758 (13)	0.0427 (4)	
C5	0.24772 (9)	0.1542 (3)	0.81301 (13)	0.0428 (4)	
H5	0.2214	0.0838	0.7446	0.051*	
C6	0.15903 (10)	−0.1584 (3)	0.89660 (15)	0.0464 (4)	
H6A	0.1831	−0.3231	0.9338	0.056*	
H6B	0.1440	−0.2005	0.8162	0.056*	
C7	0.07804 (9)	−0.0748 (3)	0.95097 (13)	0.0376 (3)	
C8	0.05197 (9)	−0.2024 (3)	1.04813 (12)	0.0388 (3)	
C9	0.02329 (9)	0.1316 (3)	0.90476 (12)	0.0388 (3)	
F1	0.10088 (7)	−0.4083 (2)	1.09813 (8)	0.0566 (3)	
F2	0.04382 (6)	0.2659 (2)	0.80981 (8)	0.0549 (3)	
N1	0.22433 (8)	0.0614 (3)	0.90663 (12)	0.0445 (3)	
S1	0.37524 (3)	0.47708 (10)	0.92848 (4)	0.05398 (19)	

### Atomic displacement parameters (Å^2^)

**Table:**

	*U*^11^	*U*^22^	*U*^33^	*U*^12^	*U*^13^	*U*^23^
C1	0.0407 (9)	0.0496 (10)	0.0913 (15)	−0.0038 (8)	0.0063 (9)	−0.0024 (10)
C2	0.0532 (10)	0.0521 (10)	0.0809 (14)	0.0022 (9)	0.0229 (9)	0.0114 (10)
C3	0.0513 (10)	0.0576 (11)	0.0517 (10)	0.0049 (8)	0.0108 (8)	0.0013 (8)
C4	0.0354 (7)	0.0451 (9)	0.0477 (9)	0.0048 (6)	0.0052 (6)	−0.0031 (7)
C5	0.0349 (7)	0.0481 (9)	0.0453 (9)	0.0027 (7)	0.0031 (6)	−0.0075 (7)
C6	0.0391 (8)	0.0437 (9)	0.0575 (10)	−0.0013 (7)	0.0107 (7)	−0.0063 (7)
C7	0.0327 (7)	0.0371 (8)	0.0427 (8)	−0.0012 (6)	0.0024 (6)	−0.0045 (6)
C8	0.0366 (7)	0.0355 (8)	0.0429 (8)	0.0042 (6)	−0.0033 (6)	0.0009 (6)
C9	0.0409 (8)	0.0389 (8)	0.0365 (8)	−0.0034 (6)	0.0030 (6)	0.0009 (6)
F1	0.0541 (6)	0.0549 (6)	0.0601 (6)	0.0183 (5)	0.0013 (5)	0.0136 (5)
F2	0.0608 (6)	0.0565 (6)	0.0492 (6)	0.0035 (5)	0.0144 (4)	0.0145 (5)
N1	0.0341 (7)	0.0488 (8)	0.0510 (8)	−0.0003 (6)	0.0056 (6)	−0.0053 (6)
S1	0.0457 (3)	0.0584 (3)	0.0562 (3)	−0.00403 (19)	−0.0039 (2)	−0.0020 (2)

### Geometric parameters (Å, º)

**Table:**

C1—C2	1.350 (3)	C6—N1	1.469 (2)
C1—S1	1.713 (2)	C6—C7	1.515 (2)
C1—H1	0.9300	C6—H6A	0.9700
C2—C3	1.414 (3)	C6—H6B	0.9700
C2—H2	0.9300	C7—C8	1.389 (2)
C3—C4	1.378 (2)	C7—C9	1.391 (2)
C3—H3	0.9300	C8—F1	1.3555 (17)
C4—C5	1.454 (2)	C8—C9^i^	1.380 (2)
C4—S1	1.7202 (17)	C9—F2	1.3531 (18)
C5—N1	1.270 (2)	C9—C8^i^	1.380 (2)
C5—H5	0.9300		
			
C2—C1—S1	112.19 (15)	N1—C6—H6A	109.4
C2—C1—H1	123.9	C7—C6—H6A	109.4
S1—C1—H1	123.9	N1—C6—H6B	109.4
C1—C2—C3	112.68 (17)	C7—C6—H6B	109.4
C1—C2—H2	123.7	H6A—C6—H6B	108.0
C3—C2—H2	123.7	C8—C7—C9	115.32 (13)
C4—C3—C2	112.58 (17)	C8—C7—C6	122.69 (14)
C4—C3—H3	123.7	C9—C7—C6	121.97 (14)
C2—C3—H3	123.7	F1—C8—C9^i^	118.11 (13)
C3—C4—C5	127.50 (15)	F1—C8—C7	119.36 (13)
C3—C4—S1	110.81 (13)	C9^i^—C8—C7	122.51 (14)
C5—C4—S1	121.70 (12)	F2—C9—C8^i^	118.72 (13)
N1—C5—C4	123.20 (14)	F2—C9—C7	119.11 (13)
N1—C5—H5	118.4	C8^i^—C9—C7	122.16 (14)
C4—C5—H5	118.4	C5—N1—C6	116.10 (14)
N1—C6—C7	111.25 (13)	C1—S1—C4	91.74 (10)
			
S1—C1—C2—C3	−0.6 (2)	C6—C7—C8—C9^i^	−177.86 (14)
C1—C2—C3—C4	0.7 (2)	C8—C7—C9—F2	−179.81 (13)
C2—C3—C4—C5	−179.95 (15)	C6—C7—C9—F2	−1.4 (2)
C2—C3—C4—S1	−0.37 (19)	C8—C7—C9—C8^i^	−0.5 (2)
C3—C4—C5—N1	−172.30 (16)	C6—C7—C9—C8^i^	177.88 (14)
S1—C4—C5—N1	8.2 (2)	C4—C5—N1—C6	−177.93 (14)
N1—C6—C7—C8	−113.68 (16)	C7—C6—N1—C5	−120.34 (16)
N1—C6—C7—C9	68.1 (2)	C2—C1—S1—C4	0.36 (15)
C9—C7—C8—F1	179.22 (13)	C3—C4—S1—C1	0.02 (14)
C6—C7—C8—F1	0.8 (2)	C5—C4—S1—C1	179.62 (14)
C9—C7—C8—C9^i^	0.5 (2)		

Symmetry code: (i) −*x*, −*y*, −*z*+2.

### Hydrogen-bond geometry (Å, º)

**Table:**

*D*—H···*A*	*D*—H	H···*A*	*D*···*A*	*D*—H···*A*
C6—H6*A*···F1	0.97	2.44	2.873 (3)	107
C5—H5···F1^ii^	0.93	2.56	3.446 (2)	159
C6—H6*B*···F1^ii^	0.97	2.63	3.542 (2)	156

Symmetry code: (ii) *x*, −*y*−1/2, *z*−1/2.
